# Harmonic trap resonance enhanced synthetic atomic spin-orbit coupling

**DOI:** 10.1038/srep46756

**Published:** 2017-04-27

**Authors:** Ling-Na Wu, Xin-Yu Luo, Zhi-Fang Xu, Masahito Ueda, Ruquan Wang, L. You

**Affiliations:** 1 State Key Laboratory of Low Dimensional Quantum Physics, Department of Physics, Tsinghua University, Beijing 100084, China; 2 Department of Physics, University of Tokyo, 7-3-1 Hongo, Bunkyo-ku, Tokyo 113-0033, Japan; 3 MOE Key Laboratory of Fundamental Physical Quantities Measurements, School of Physics, Huazhong University of Science and Technology, Wuhan 430074, China; 4 Institute of Physics, Chinese Academy of Sciences, Beijing 100080, Peoples Republic of China; 5 Collaborative Innovation Center of Quantum Matter, Beijing, China

## Abstract

Spin-orbit coupling (SOC) plays an essential role in many exotic and interesting phenomena in condensed matter physics. In neutral-atom-based quantum simulations, synthetic SOC constitutes a key enabling element. The strength of SOC realized so far is limited by various reasons or constraints. This work reports tunable SOC synthesized with a gradient magnetic field (GMF) for atoms in a harmonic trap. Nearly ten-fold enhancement is observed when the GMF is modulated near the harmonic-trap resonance in comparison with the free-space situation. A theory is developed that well explains the experimental results. Our work offers a clear physical insight into and analytical understanding of how to tune the strength of atomic SOC synthesized with GMF using harmonic trap resonance.

Resonance phenomena^1^ frequently occur in Nature. When the frequency of a time-periodic external drive matches a system’s resonance, the response is dramatic. A folklore wisdom warns that soldiers crossing a bridge should not march in unison to prevent its collapsing from accidentally stepping onto the resonance. Even at multiple resonant frequencies, such as parametric resonance when the driving frequency is twice the system’s characteristic frequency, the response can still be quite substantial. In quantum mechanics, resonances become ubiquitous as a result of quantization, where stationary states of a system feature definite eigenenergies. A transition between two eigenstates is resonantly enhanced when the frequency of an external coupling matches their energy difference^2^,^3^.

This article reports our experimental observation and theoretical vindication of an enhanced atomic spin-orbit coupling (SOC) synthesized with a modulated gradient magnetic field (GMF) applied to atoms in a harmonic trap. SOC, which couples a particle’s spin to its orbital motion, constitutes one of the most important interactions in condensed matter physics. In the strong coupling regime, SOC gives rise to nontrivial topological bands, which support many exotic states and phenomena, including topological band/Mott insulator, quantum-number fractionalization and magneto-electric effects^4^,^5^. In recent years, atomic quantum gases have emerged as powerful quantum simulators for condensed matter systems^6^,^7^. Strong atomic SOC often plays crucial roles in the increasing list of desired ingredients for artificial gauge fields^8^,^9^,^10^,^11^,^12^,^13^,^14^,^15^,^16^,^17^,^18^,^19^,^20^.

The breakthrough on synthetic SOC came in 2011^13^, when Spielman’s group observed a special type of one-dimensional (1D) SOC: an equal-weighted sum of Rashba^21^ and Dresselhaus^22^ types of SOC created by the momentum-sensitive Raman coupling between two internal states of ^87^Rb atoms. Since then, the Raman scheme has become the prototype for studies involving 1D atomic SOC^23^,^24^,^25^,^26^,^27^. Recently, the observations of two-dimensional (2D) SOC relying on atom-photon interactions have been reported^28^,^29^,^30^. In the Raman scheme, the strength of synthesized SOC is limited by photon momentum transfer and constrained by Raman laser beam geometry. A protocol for tuning the strength of SOC including switching its sign through periodically modulating effective Rabi frequency^31^ in the Raman scheme has also been realized^32^.

An alternative method of creating synthetic atomic SOC is to use pulsed or time-periodic GMF^33^,^34^,^35^,^36^,^37^,^38^,^39^, which can be implemented free from atomic spontaneous emission. Its underlying mechanism is the Stern-Gerlach effect, whereby the periodic GMF imparts a spin-dependent momentum impulse to the atomic center-of-mass motion. This spin-dependent impulse can be described in terms of the same linear coupling between the spin (or pseudo-spin) with the atomic orbital (center-of-mass) motion as in the Raman scheme. Through concatenating GMF pulses along two orthogonal directions, genuine Rashba, Dresselhaus, or even arbitrary types of SOC in 2D can be synthesized for atoms with arbitrary hyperfine spins^33^,^34^,^35^. Very recently, essential features demonstrating 1D tunable SOC synthesized with a periodic modulated GMF have been reported^38^.

This work presents a different method for controlling the strength of atomic SOC synthesized with GMF by making use of harmonic trap resonance for atomic center-of-mass motion. It is beyond the straightforward scheme of tuning the strength of momentum impulse as demonstrated recently^38^. Furthermore, it differs from the reported tuning scheme^32^ based on the amplitude-modulated Raman coupling^31^, which can only decrease the strength of SOC. The harmonic trap resonance scheme we report here opens a different avenue for reaching the strong SOC regime.

## Results

The experiment is inspired by the success of synthesizing atomic SOC from a time-periodic GMF^34^,^38^. Specifically, under a periodically modulated 1D GMF, atomic center-of-mass experiences a spin-dependent force, whose overall effect is simply to shift the momentum from *p*_*x*_ to 
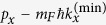
 for the atomic spin component *m*_*F*_, where 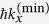 denotes the minimum of the modified dispersion curve. The momentum of the equilibrium state is thus translated by a spin-dependent amount 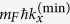, which is equivalent to a synthetic SOC with strength 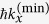. Experimentally, this effective strength of SOC is determined from the measured displacement of the atomic cloud.

**Figure 1:**
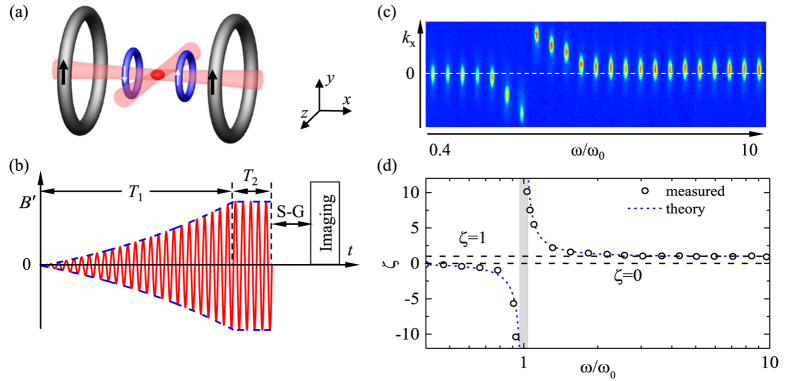
Harmonic-trap-resonance enhanced SOC. (**a**) Schematic illustration of the experimental setup, consisting of the bias (gray) and gradient (blue) magnetic coils. The condensate (red football shape) is produced at the center of a crossed optical dipole trap formed from laser beams in pink. Its location coincides with the center of the gradient coil configuration. (**b**) Time sequence of our experiments. The modulation amplitude (delimited by the blue dashed envelop) of the gradient magnetic field *B*′(*t*) (shown in red) is adiabatically ramped up to an effective value corresponding to *k*_so_ = 1.25 *μm*^−1^ within *T*_1_ and held on for *T*_2_, followed by the Stern-Gerlach (S-G) separation before absorption imaging. To ensure adiabaticity during the ramp, *T*_1_ = 250 ms and *T*_2_ = 50 ms are chosen for the modulation frequency *ω* > (2*π*)100 Hz and *T*_1_ = 25*τ* and *T*_2_ = 5*τ*, with *τ* = 2*π/ω* being the modulation period, for *ω* < (2*π*)100 Hz. (**c**) Absorption images for the momentum-shifted atomic clouds in the |*m*_*F*_ = −1〉 state at different values of *ω*. Darker red denotes higher optical density. The abscissa is not to scale. Each measured off-set atomic cloud corresponds to a data point shown in (**d**) in the same order of increasing modulation frequency from left to right. The dashed line denotes *k*_*x*_ = 0 for without GMF or SOC. (**d**) The measured values of the scaled SOC strength *ζ* (black open circles) as a function of *ω*, which agree perfectly with Eq. (8) shown in the blue dotted curve. In the shaded band region surrounding the trap resonance, the driven atomic cloud fails to adiabatically reach the momentum-shifted equilibrium state after *T*_2_.

A typical experiment starts with a ^87^Rb condensate of 1.2 × 10^5^ atoms in the state 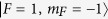 confined inside a crossed dipole trap whose minimum potential region is approximately harmonic with frequencies (*ω*_*x*_, *ω*_*y*_, *ω*_*z*_) = 2*π* × (77, 136, 77) Hz along three orthogonal spatial directions *x, y*, and *z*. The 1D GMF is implemented by a combination of a 3D quadrupole magnetic field 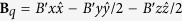 and a 5.7 Gauss bias field 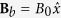 [Fig. 1(a)], whose linear and quadratic Zeeman shifts correspond to (2*π*)4 MHz and (2*π*)2.34 kHz, respectively. More details about the magnetic field control is as described in ref. 38. The amplitude for the GMF is sinusoidally modulated as *B*′(*t*) = 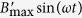, which translates into a 1D SOC strength 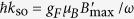, where *g*_*F*_ denotes the Landé g-factor and *μ*_*B*_ the Bohr magneton, provided the modulation frequency *ω* is far away from trap resonance, as confirmed in a recent experiment^38^.

As shown in Fig. 1(b), the |*m*_*F*_ = −1〉 condensate is loaded into the momentum-shifted equilibrium state by adiabatically ramping up the GMF modulation amplitude to a value corresponding to the SOC strength of *k*_so_ = 1.25 *μm*^−1^ within 250 ms (or 25 modulation periods for *ω* < (2*π*)100 Hz), and then held on for another 50 ms (or 5 modulation periods for *ω* < (2*π*)100 Hz). At integer multiples of the modulation period *τ* = 2*π/ω*, the crossed dipole trap is turned off in less than 10 *μ*s. Subsequently, the condensate expands for about 24 ms, during which different Zeeman components are Stern-Gerlach separated by an inhomogeneous magnetic field along the vertical direction. A bimodal fit to the atomic cloud density profile measured through the standard absorption imaging, as shown in Fig. 1(c), yields the shifted center-of-mass position for the condensate. The spatial displacement from that without SOC is used to derive the momentum shift 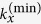, from which the scaled SOC strength *ζ* = 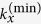/*k*_so_ is computed.

A clear resonance behavior is observed for *ζ*, as shown in Fig. 1(c) and (d), for its dependence on the modulation frequency *ω* relative to the trap frequency *ω*_0_ = *ω*_*x*_ = (2*π*)77 Hz. Above the trap frequency *ω*_0_, *ζ* increases with decreasing *ω*, from *ζ* = 1 for *ω* far above the resonance to a peak when *ω* approaches *ω*_0_. Below the trap frequency, *ζ* changes its sign, with its magnitude growing from *ζ* = 0 for *ω* far below the resonance to a peak around the resonance. The enhanced response on the opposite sides of the resonance is out of phase as a result of the *π* phase shift across a resonance. Limited by our present setup, we operate in the regime of small momentum impulse and observe nearly ten-fold enhancement for *ζ* at *ω/ω*_0_ = 1.03, where heating remains insignificant. The effect of heating-induced damping becomes noticeable in the immediate vicinity of resonance.

## Discussion

The dramatic resonance enhancement of SOC due to the harmonic trap cannot be explained by the previous theory for atoms in free space^34^, which neglects the influence of the trapping potential on atomic motion. One might have naively concluded that an analogous calculation that incorporates the effect of the trap potential into the previously studied free-space model would find the agreement with the observed resonance. Unfortunately, this is easily said than done. To demonstrate it, we briefly recapitulate the basic idea of the previous theory^34^. For an atom of mass *m* in free space and in the presence of a sinusoidally modulated GMF along the *x*-direction, the effective 1D Hamiltonian is given by


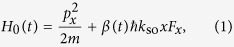


where *p*_*x*_ is the momentum of an atom, *F*_*x*_ is the *x*-component of its spin *F*, and *β(t*) = *ω*sin(*ωt*) is the temporal profile for the coupling strength between the time-dependent GMF and atomic magnetic dipole moment measured in units of modulation amplitude *ħk*_so_. The Schrödinger equation for *H*_0_(*t*) of Eq. (1) can be more easily handled if we introduce a unitary transformation 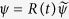 with 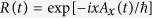, which corresponds to a momentum translation by the spin-dependent impulse 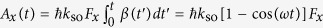 from the GMF. The wave function 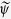 in the rotating frame is then governed by the momentum shifted Hamiltonian 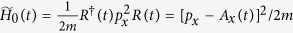, which commutes with itself at different times, 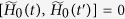. The corresponding time evolution operator takes a simple form 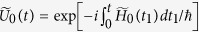. After one period of evolution *τ* = 2*π/ω*, we obtain *A*_*x*_(*τ*) = 0, or *R(τ*) = 1. Hence, the wavefunctions in the two frames coincide, 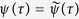, and the effective Hamiltonian for the whole period is given by


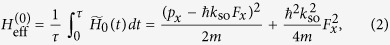


where the first term describes the SOC of strength *ħk*_so_ and the second term acts like a quadratic Zeeman shift.

In the presence of a 1D harmonic trap *V*_trap_ = *mω*_0_^2^*x*^2^/2, the Hamiltonian changes into


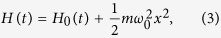


which in the rotating frame becomes


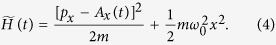


Unlike the case of a free atom discussed above, the two 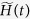
’s in Eq. (4) at different times do not always commute due to the presence of *V*_trap_. The corresponding unitary evolution operator then takes a more general form 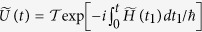, where 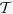 denotes time ordering. This unitary evolution operator takes such a complicated form that it is difficult to derive the effective Hamiltonian in a straightforward manner. Therefore, we have to resort to other means for a compact solution capable of explaining the resonant behavior observed.

We note that Hamiltonian (3) also describes a sinusoidally driven harmonic oscillator, whose quantum-mechanical propagator can be obtained in the explicit analytic form. Hence, we can get the effective Hamiltonian of the system by making use of the propagator. For Hamiltonian (3), its propagator is given by (see Methods).


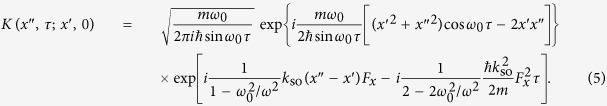


The effective Hamiltonian of the system should give the same propagator as Eq. (5). Without loss of generality, it is reasonable to infer that the effective Hamiltonian will not be much different from that in free space in Eq. (2). We therefore assume


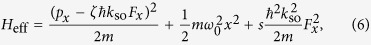


where *ζ* and *s* denote modifications to the strength of SOC and the quadratic Zeeman shift, respectively, when the trap potential *V*_trap_ is present. The corresponding propagator in this case is found to be (see Methods)


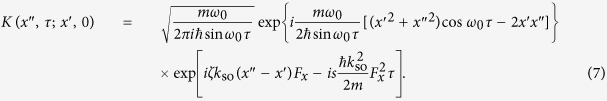


The equivalence between the two propagators of Eqs. (5) and (7) thus gives


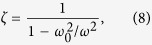


and 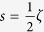. In other words, the effective Hamiltonian for atoms in a harmonic trap driven by a sinusoidally modulated GMF is found to be given by Eq. (6), which is similar in form to the case of a free atom except for *ζ* and *s* in Eq. (8).

The factor *ζ* in Eq. (8) is plotted as the blue dotted curve in Fig. 1(d), which is found to agree well with the measured data except in the immediate vicinity of the trap resonance [shaded band region in Fig. 1(d)], where the finite lifetime of the condensate makes it difficult to reach equilibrium. It clearly shows that as the modulation frequency *ω* approaches the trap frequency *ω*_0_, the SOC becomes enhanced. More specifically, when approaching the resonance from above, the effective SOC is increasingly enhanced. Upon crossing the resonance *ω*_0_, the effective SOC reverses its sign, and the factor *ζ* gradually decreases and eventually tails off to zero at frequencies much smaller than *ω*_0_. This dependence of *ζ* on the modulation frequency highlights the tunability discussed in this work. In the immediate vicinity of the resonance, the amplitude of atomic micro-motion due to periodic modulation is so large that the Gaussian-shaped optical trap cannot be well approximated by a harmonic trap anymore, and as a result of the large amplitude oscillations the condensate collapses.

The observed enhancement of SOC is reminiscent of the resonance phenomenon in a driven harmonic oscillator. The factor *ζ* shown in Eq. (8) can be directly verified as well by comparing the equations of motion for the periodically-driven Hamiltonian (3) with the effective Hamiltonian form of (6) (see supplementary material). While the factor *s* cannot be derived in such a way. When the atomic spin is prepared into a superposition state, the seemingly classical driven equation of motion becomes spin dependent, a situation without a classical analog. For the 1D case considered here, the synthesized SOC can be gauged away by making the transformation 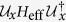 with 
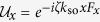
. However, when tracking the dynamics for the different atomic spin components, the accumulated phase from the SOC term, is real as confirmed in the recent experiment^38^. Thus, although the effective SOC we describe can be gauged away, the observation gives gauge dependent results which implicate the presence of synthetic SOC. Furthermore, in the presence of a uniform bias magnetic field, which gives rise to an interaction ∝ *F*_*z*_, or when any other non-commuting interactions are present, the synthesized SOC discussed above persists and cannot be gauged away even in 1D system^38^,^40^.

In conclusion, for the atomic SOC synthesized from a time-periodic GMF, we observed a resonant behavior which highlights nearly ten-fold enhanced SOC when the modulation frequency is close to but higher than the trap frequency. This resonance is accompanied by a progression towards vanishing SOC on the lower modulation frequency side and a reduction to the value for a free atom in the higher modulation frequency side. We develop a theory that well explains the experimentally observed resonant behavior. Compared with atoms in free space under a sinusoidally modulated GMF, we find that an effective SOC Hamiltonian for atoms confined inside a harmonic trap takes an analogous form, except for a frequency-dependent prefactor. This prefactor reveals the resonant behaviour as the periodic drive hits the motional resonance of the harmonic trap.

## Methods

### Propagator of a forced 1D harmonic oscillator

The quantum-mechanical propagator *K(x*″, *t*″; *x*′, *t*′) describes the transition amplitude from one space-time position (*x*′, *t*′) to another (*x*″, *t*″). For a time-dependent driven harmonic oscillator described by Hamiltonian


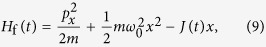


the propagator is given by ref. 41


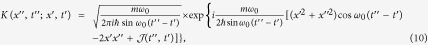


where


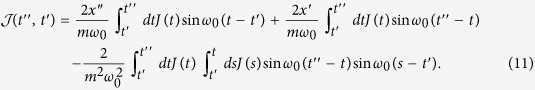


For the example considered in the main text, we have *J(t*) = −*β(t)ħk*_so_*F*_*x*_ and 
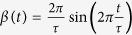
, and therefore we find


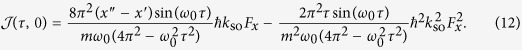


Thus, the propagator for a sinusoidally driven harmonic oscillator is given by Eq. (5) in the main text.

### Propagator for the momentum-shifted 1D harmonic oscillator

To obtain the propagator for a momentum-shifted harmonic oscillator described by the effective Hamiltonian (6) in the main text, we first derive the propagator *K*_t_ for the unitary transformed Hamiltonian


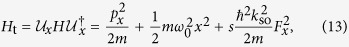


with 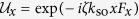. Based on the propagator for the harmonic oscillator,


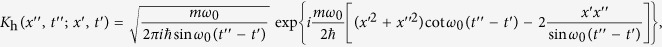


which is easily reproduced if we put 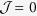 in Eq. (10) and making use of the properties for the propagators, we find that


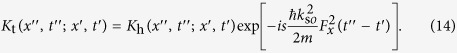


Therefore, by making inverse unitary transformation, we obtain the propagator for the effective Hamiltonian (6) as


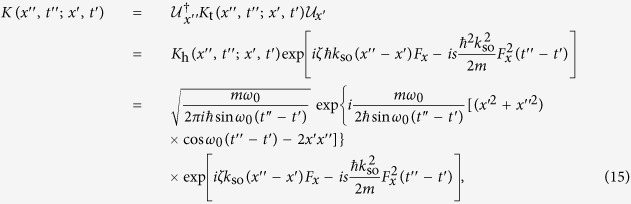


or Eq. (7) in the main text.

## Additional Information

**How to cite this article:** Wu, L.-N. *et al*. Harmonic trap resonance enhanced synthetic atomic spin-orbit coupling. *Sci. Rep.* **7**, 46756; doi: 10.1038/srep46756 (2017).

**Publisher's note:** Springer Nature remains neutral with regard to jurisdictional claims in published maps and institutional affiliations.

## Supplementary Material

Supplementary Material
