# Supplementary material for: Harmonic trap resonance enhanced synthetic atomic spin-orbit coupling
Source: Sci Rep. 2017 Apr 27;7:46756. doi: 10.1038/srep46756 (PMC5406833; doi:10.1038/srep46756)
Supplement: Supplementary Material [file srep46756-s1.pdf]

Supplementary material: Harmonic trap resonance enhanced synthetic atomic spin-orbit coupling

Ling-Na Wu,[1] Xin-Yu Luo,[1] Zhi-Fang Xu,[2,3] Masahito Ueda,[2] Ruquan Wang,[4,5,*] and L. You[1,5,†]

[1] *State Key Laboratory of Low Dimensional Quantum Physics, Department of Physics, Tsinghua University, Beijing 100084, China*
[2] *Department of Physics, University of Tokyo, 7-3-1 Hongo, Bunkyo-ku, Tokyo 113-0033, Japan*
[3] *MOE Key Laboratory of Fundamental Physical Quantities Measurements, School of Physics, Huazhong University of Science and Technology, Wuhan 430074, China*
[4] *Institute of Physics, Chinese Academy of Sciences, Beijing 100080, Peoples Republic of China*
[5] *Collaborative Innovation Center of Quantum Matter, Beijing, China*

(Dated: March 9, 2017)

PACS numbers: 67.85.De, 03.75.Mn, 67.85.Jk

* ruquanwang@aphy.iphy.ac.cn
† lyou@mail.tsinghua.edu.cn

In this supplementary material, we give the derivation of the enhancement factor [Eq. (8) in the main text] through the equation of motion.

For the time-dependent Hamiltonian [Eq. (3) in the main text]

$$H\left(t\right)=\frac{p_x^2}{2m}+\omega\sin\left(\omega t\right)\hbar k_{\mathrm{so}}xF_x+\frac{1}{2}m\omega_0^2x^2, \tag{S1}$$

the equation of motion is given by

$$m\ddot{x}+\omega\sin\left(\omega t\right)\hbar k_{\mathrm{so}}F_x+m\omega_0^2x=0. \tag{S2}$$

Under the initial condition of $x(0)=0$ and $\dot{x}(0)=p_x(0)/m=0$, its solution reads

$$x\left(t\right)=\frac{\hbar k_{\mathrm{so}}}{m\omega_0}F_x\frac{\omega}{\omega^2-\omega_0^2}\left[\omega_0\sin\left(\omega t\right)-\omega\sin\left(\omega_0t\right)\right]. \tag{S3}$$

At integer multiples of the modulation period, i.e., $t=n\tau=n\cdot2\pi/\omega$, it reduces to

$$x\left(n\tau\right)=-\frac{\hbar k_{\mathrm{so}}}{m\omega_0}F_x\frac{\omega^2}{\omega^2-\omega_0^2}\sin\left(n\omega_0\tau\right). \tag{S4}$$

On the other hand, for the effective Hamiltonian [Eq. (6) in the main text]

$$H_{\mathrm{eff}}=\frac{\left(p_x-\zeta\hbar k_{\mathrm{so}}F_x\right)^2}{2m}+\frac{1}{2}m\omega_0^2x^2+s\frac{\hbar^2k_{\mathrm{so}}^2}{2m}F_x^2, \tag{S5}$$

the equation of motion is given by

$$\ddot{x}+\omega_0^2x=0. \tag{S6}$$

Under the initial condition of $x(0)=0$ and $\dot{x}(0)=[p_x(0)-\zeta\hbar k_{\mathrm{so}}F_x]/m=-\zeta\hbar k_{\mathrm{so}}F_x/m$, its solution reads

$$x\left(t\right)=-\zeta\frac{\hbar k_{\mathrm{so}}}{m\omega_0}F_x\sin\left(\omega_0t\right). \tag{S7}$$

At integer multiples of the modulation period, i.e., $t=n\tau$, it becomes

$$x\left(n\tau\right)=-\zeta\frac{\hbar k_{\mathrm{so}}}{m\omega_0}F_x\sin\left(n\omega_0\tau\right). \tag{S8}$$

Comparison of Eqs. (S4) and (S8) then gives the enhancement factor

$$\zeta=\frac{\omega^2}{\omega^2-\omega_0^2}, \tag{S9}$$

which is the same as Eq. (8) in the main text derived from the quantum propagator approach.
